# Medical expenses of patients with Favism admitted to 17th Shahrivar Hospital compared to G6PD enzyme screening cost, in north of Iran

**Published:** 2014-04-20

**Authors:** B Darbandi, M Noghbaei, F Mehrabian, M Jafroodi

**Affiliations:** 1 Pediatric Hematologist and Oncologist,: Pediatrics Growth Disorders Research Center, 17th Sharivar Hospital, Guilan University of Medical Sciences, Rasht, Iran; 2 Pediatrician, 31thKhordad Hospital, Manjil, Iran; 3 Health Service Management,Guilan University of Medical Sciences. Rasht,Iran

**Keywords:** Favism, Cost of disease, Screening, G6PD

## Abstract

**Background:**

Glucose-6-phosphate dehydrogenase (G6PD) enzyme deficiency is one of the prevalent disorders in Guilan province, northern Iran, causing many patients to suffer from acute hemolysis. This disease has imposed tremendous costs both on patients and Health systems.

The aim of this study was to compare the direct costs of favism treatment on patients and health system with G6PD enzyme screening test.

**Materials and Methods:**

In this descriptive prospective study, the medical and hospital costs of acute hemolysis due to G6PD deficiency were calculated and compared with the expenses of screening newly born infants for this disorder in Rasht. Data was collected by a questionnaire.Student’s t-Test and chi-squared test were recruited and data was analyzed using SPSS ver. 20.

**Results:**

In this study, 101 hospitalized patients with favism (72 male and 29 female) admitted from October 2011 to the end of September 2012 were included. The average cost of treatment for these patients was approximately 726000000Rials (for each patient 7190000 Rials), which was about half of the cost of screening for all newborn infants in Rasht during this period.

**Conclusion:**

The cost of G6PD enzyme screening in Rasht is substantially lower than the cost of treating hospitalized patients with Favism.

## Introduction

Glucose-6-phosphate dehydrogenase (G6PD) deficiency is the most prevalent red blood cell enzyme deficiency in human being which is estimated to afflict about 400000000 people worldwide with the global prevalence of 4.6%(1). In areas with endemic malaria, the deficiency prevalence increases 5 to 25%. Several epidemic studies have recorded 62% prevalence in Middle East (especially in Kurdish Jews) and 31% in North Vietnam(2,3). Moreover, the incidence of this disorder is high in certain subgroups in North America especially in blacks, eastern Asia emigrants, Southern Asia, Greece and Italy (4). General incidence of G6PD deficiency in Iran is estimated to be 10 to 14% (4). In a study conducted in Shiraz, 6% of male donors were afflicted to this deficiency without being aware of it(5). In Isfahan the rate of incidence was 3.2% as determined by another study (6) .It is worth noting that the highest frequency of Favism has occurred in north of Iran as to be 27%(7).Another screening study on infants reported 6.4% incidence of the disease in Guilan (8).

Favism is a symptomatic G6PD deficiency of enzyme which occurs after consumption of fresh broad beansthat contains convicine, isouramil and divicine resulting finally in the production of hydrogen peroxide and other oxygen-causing reactive compounds.

The peak of Favism outbreak happens in April till May.Frozen or cooked beans intake also leads to hemolysis as seen in Chinese snacks. Also, hemolysis due to G6PD deficiency has been found in infants whose mothers consume beans. Favism most often occurs in 1 to 5year old boys. The symptoms appear generally 5 to 24 hours after beans consumption-occasionally after 24 to 48 hours or more, include pale, Jaundice, and darkness of the urine. Fever, headache, nausea and backache (9).

Upon the beginning of acute hemolysis, hemoglobin and hematocrit quickly decline and if the attack is severe, binding protein to free hemoglobin like haptoglubin will be saturated so that free hemoglobin in plasma and subsequently in urine appears. Making diagnosis is based on direct or indirect confirmation of red blood cells G6PD activity reduction. Direct measurement reveals that G6PD activity in patients with Favism is 10% or less than that of a normal person (9).

Treatment is given according to the clinical symptoms and prevention of hemolysis is the most important measure.

Because of high prevalence and subsequent complications, infant screening programs have been established in non-western countries such as central Asia, Eastern Europe and southern Asia (9).Regarding to expenses, comparative studies showed that cost –effectiveness in some countries was in favor of screening while in some others in favor of treatment.

Due to similarity of the epidemiological characteristics of the disease in north part of Iran (Caspian Sea coast) with the Mediterranean areas and south and east of Asia, the prevalence of G6PD seems to be high here(10).Thus, regarding to geographical location and increasing referrals of children with acute hemolysis due to intake of beans in warm season of year or due to oxidant consumption like drugs (11) , we decided to calculate hospital and medical expenses of patients with Favism during one year in 17thSharivar hospital and compare it with the cost of screening test of all newborn children during the same period. If a significant difference could be found, then performing screening program for all newborn babies in this area is recommended.

## Materials and Methods

In order to determine direct treatment cost of patients with G6PD deficiency, a prospective descriptive study was carried out. By consensus sampling, all patients with Favism admitted from October2011 to September 2012 due to acute hemolysis from G6PD deficiency were enrolled. All patients were visited and data such as age on admission, gender, number of transfusion of packed cells, days of hospital stay, and treatment cost were recorded in a standard questionnaire by a researcher. Patients with hemolysis from other causes were excluded. 

**Statistical Analysis**

Collected data were analyzed using SPSS ver.20 and comparison of the number of hospital stay days and blood transfusions with cost of hospitalization were performed by Student’s t-Test.

## Results

During the study period, 101 patients-72 male (71%) and29 (29%) female-were included. The gender ratio of male to female was 2.4 to 1. In diagram1 different range ages of children with Favism is illustrated with a peak in 2 to 4 years old. The mean number of transfusions was 2.45±2.8 and 53.5% of patients received transfusion 2 times (diagram2). The mean time of hospital stay was 3.23±1.04 days in that one day was the least and 6 days was the most. Most of the patients -42 cases (41.6%) - stayed 3 days (diagram 3). The most referrals to hospital occurred in spring [80 cases (79.2%)(diagram4)]. The mean cost that each patient should pay upon discharge was 1084000±167000 Rials which for all patients with Favism during one year is 109484000 Rials. Also each unit of packed red cells costs 100 dollars or 2450000 Rials( one dollar equals about 24500 Rials) and since 247 units are needed for all of these patients during one year, the total cost will be 730000000 Rials-approximately 30000 dollars. It should be mentioned that the above figures are only direct cost of hospital stay and provision and injection of packed red cells and indirect cost was not estimated due to difficulties in calculation. On the other hand, nearly 10720 children are born each year in Rasht and if G6PD screening program is carried out on all of them, the total cost will be 405000000 Rials, about 16500 dollars – the cost of each G6PD enzyme test is 37800 Rials-that is approximately 45% less than the direct cost of all patients during one year.

**Figure 1:**
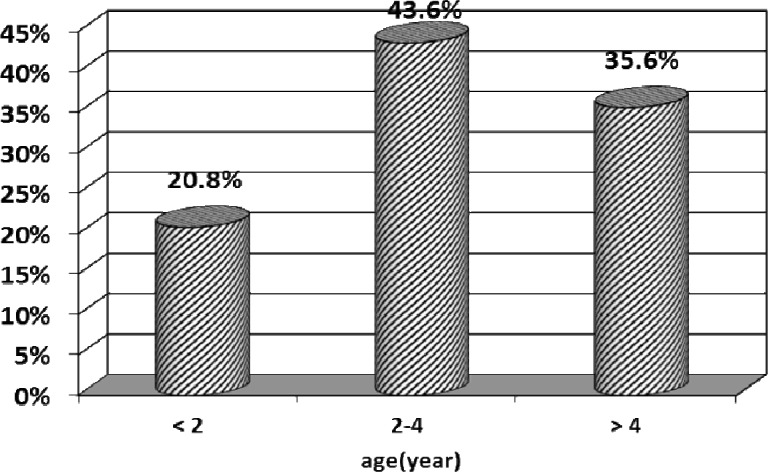
age relative frequency in patients

**Figure 2:**
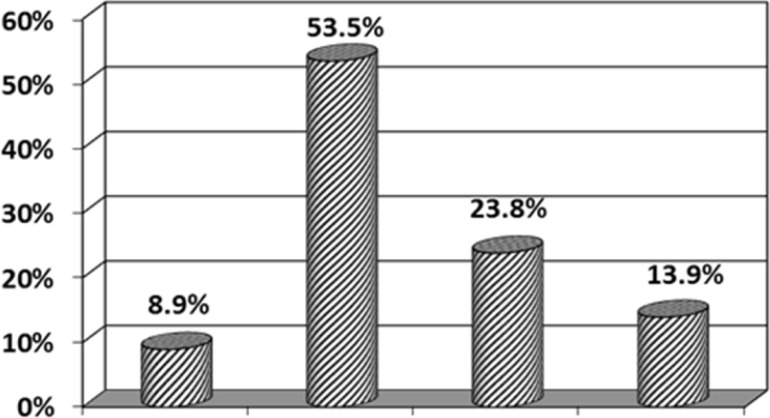
packed cell transfusion relative frequency

**Figure 3:**
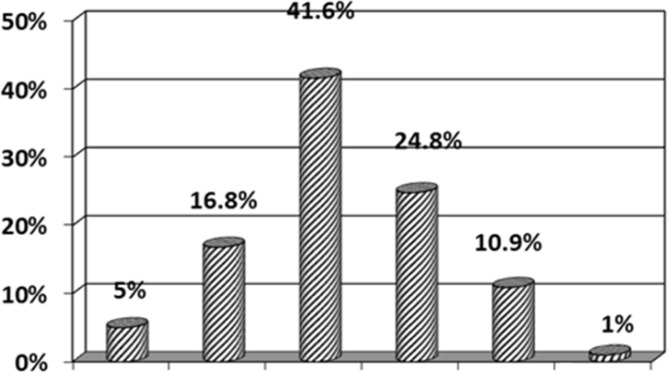
hospitalization day frequency

**Figure 4:**
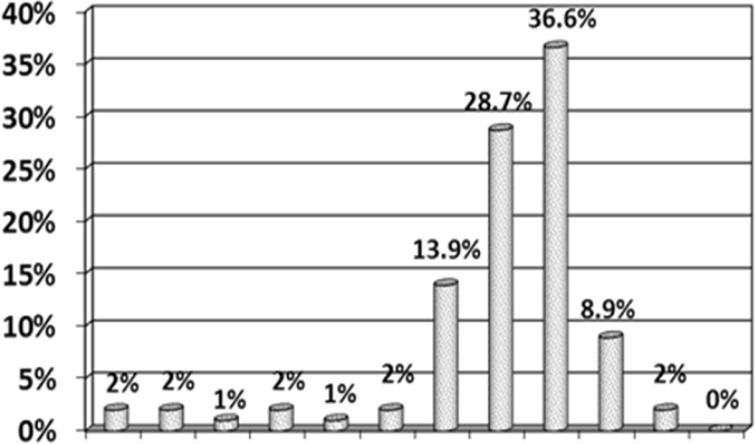
frequency of hospitalization in terms of months of the year

## Discussion

Our findings revealed that during a year, 101 children with Favism admitted to the hospital so that 247 units of packed cell were used and 326 day-tables were occupied. About 79.2% of them referred to hospital during spring which shows a season-dependent outbreak which should be considered in terms of procurement, tables and treatment planning in the hospital.

All patients were treated according to the treatment protocol in the hospital. All of the patients needed at least one transfusion of packed cell as well as serum therapy and appropriate tests. The mean number of packed cell transfusion for each patient was 2.43 and the mean time for hospital stay was 3.23 days. 

 Patients’ medical expenditure consisted of two parts; first, total expenses of hospitalization including diagnostic and treatment cost (as recorded in the patients bill forms); second, packed cell transfusion expenditure which makes most part of the overall cost and ( based on hospital regulations) is not paid by the patients with Favism. Therefore, aside from indirect cost and burden of the disease, the direct cost for these patients was 726000000 Rials.

In order to compare medical expenses with G6PD screening cost, the mean number of all new born children in Rasht during 5 last years was obtained from Rasht office of Registration and Records and the rate was determined to be 10725 per year. On the other hand, the cost of G6PD screening test for each neonate is 378000 Rials and for all neonates in one year is 405000000 Rials which is about 55% of the direct hospital cost. It should be mentioned that if indirect costs of Favism such as the missed days of work, the parents, transportation cost, incidence of convulsion, kidney failure and emotional stress were included, the cost of hemolysis attack would be much more than screening test expenses.

## Conclusion

Regarding to high prevalence of G6PD deficiency in Rasht and other north parts of Iran that impose tremendous treatment expenditure to patients and health care systems, performing screening test for activity of this enzyme is recommended.

## Conflict of interest

The authors have no conflict of interest.
